# SPA^H^M(a,b): Encoding the Density Information from Guess Hamiltonian in Quantum Machine Learning Representations

**DOI:** 10.1021/acs.jctc.3c01040

**Published:** 2024-01-16

**Authors:** Ksenia R. Briling, Yannick Calvino Alonso, Alberto Fabrizio, Clemence Corminboeuf

**Affiliations:** † Laboratory for Computational Molecular Design, Institute of Chemical Sciences and Engineering, École Polytechnique Fédérale de Lausanne, 1015 Lausanne, Switzerland; ‡ National Centre for Computational Design and Discovery of Novel Materials (MARVEL), École Polytechnique Fédérale de Lausanne, 1015 Lausanne, Switzerland

## Abstract

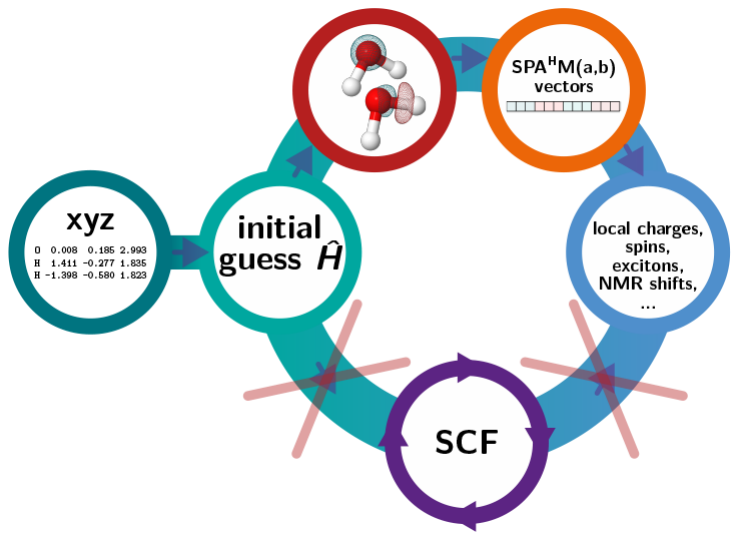

Recently, we introduced a class of molecular representations for kernel-based regression methods—the spectrum of approximated Hamiltonian matrices (SPA^H^M)—that takes advantage of lightweight one-electron Hamiltonians traditionally used as a self-consistent field initial guess. The original SPA^H^M variant is built from occupied-orbital energies (i.e., eigenvalues) and naturally contains all of the information about nuclear charges, atomic positions, and symmetry requirements. Its advantages were demonstrated on data sets featuring a wide variation of charge and spin, for which traditional structure-based representations commonly fail. SPA^H^M(a,b), as introduced here, expand the eigenvalue SPA^H^M into local and transferable representations. They rely upon one-electron density matrices to build fingerprints from atomic and bond density overlap contributions inspired from preceding state-of-the-art representations. The performance and efficiency of SPA^H^M(a,b) is assessed on the predictions for data sets of prototypical organic molecules (QM7) of different charges and azoheteroarene dyes in an excited state. Overall, both SPA^H^M(a) and SPA^H^M(b) outperform state-of-the-art representations on difficult prediction tasks such as the atomic properties of charged open-shell species and of π-conjugated systems.

## 1 Introduction

Physics-based machine learning representations, also known as representations for quantum machine learning (QML),^1−5^ are rooted in the fundamental principle that all the (static) information about a neutral chemical system is uniquely encoded into the system-specific parameters that fix the electronic Schrödinger equation: nuclear charges {*Z*_*I*_} and positions {**R**_*I*_}. Owing to their physical origins, these representations are highly general and have a deep connection to quantum-chemical targets. Hence, they have been broadly exploited to supply fast and accurate predictions of a myriad of atomistic chemical properties.

To ensure efficient predictions, most QML representations encode the information relative to the atoms and their environment through the derivation of rather simple nonlinear functions of {*Z*_*I*_} and {**R**_*I*_}, thus bypassing the construction of the Hamiltonian entirely. Most popular examples include representations built from internal coordinates (MBTR,^6^ PIPs,^7−11^ and graph-based representations^12^), those that encode regions of atomic geometries by using a local expansion of a Gaussian smeared atomic density [Behler–Parrinello symmetry functions,^13−15^ smooth overlap of atomic positions (SOAP),^16,17^ the overlap fingerprint,^18^ NICE,^19^ and ACE^20−22^], as well as those based on values or fingerprints of physically inspired potentials [Coulomb matrix,^23,24^ bag of bonds,^25^ (a)SLATM,^26^ LODE,^27^ FCHL18,^28^ and FCHL19^29^].

Each of these categories of representations have led to impressive performances for the predictions of both prototypical and complex molecular or material properties^30^ such as atomization energies,^23^ multipole moments,^31^ polarizabilities,^17,32^ HOMO–LUMO gaps,^33,34^ molecular forces,^35−37^ potential energy surfaces,^13,38,39^ electron densities,^40−43^ density functionals,^44^ and many-body wave functions.^45^ Yet, since such representations are functions of *Z*_*I*_ and **R**_*I*_ only, achieving the same level of accuracy for chemical targets inherently dependent upon changes in electron delocalization, spin, or charge remains a challenge and additional electronic information (i.e., the Hamiltonian) is needed. An alternative approach consists in adding one more layer between the geometry and the representation and complementing the latter with some quantum-chemical information computed from the former. Illustrative examples include OrbNet,^46,47^ which uses quantum-mechanical operators obtained from a converged semiempirical computation as input features for a neural network, as well as methodologies such as EHML-ML^48^ and DFTB-ML^49^ aiming at refining the parameters characteristic of semiempirical methods (e.g., Hückel theory and DFTB) to achieve higher-level accuracy. Alternative models such as EPNN^50^ propose a heuristic neural-network-based partitioning scheme to provide fast and reliable quantum-like atomic charges as input for predictive models. AIMNet^51^ with the neural spin-charge equilibration unit^52^ takes {**R**_*I*_}, {*Z*_*I*_}, and total molecular charge and spin multiplicity to learn a state-specific representation with a message-passing neural network. More computationally demanding alternatives consist in featurizing components of fully converged Hartree–Fock-level matrices, operators, densities, or determinants, as in DeePHF,^53^ DeePKS,^54^ MO-ML,^55−57^ the orbital-based FJK representation,^58^ and the kernel density functional approximation^59^ (KDFA). Also relevant to this category is the recent introduction^60^ of Coulomb lists and smooth overlap of electron densities that bridge geometry-based descriptors with electronic structure theory. The recently introduced matrix of orthogonalized atomic orbital coefficients proposes a compact although more expensive representation derived from an orbital localization scheme.^61^

With the same purpose of encoding valuable electronic information, we recently introduced the spectrum of approximated Hamiltonian matrices (SPA^H^M) representations family,^62^ which has the advantage of avoiding the self-consistent field (SCF) procedure. Specifically, the eigenvalue SPA^H^M (ε-SPA^H^M) is a compact global representation consisting of occupied-orbital eigenvalues extracted from lightweight one-electron Hamiltonians traditionally used as an SCF initial guess in molecular quantum chemistry codes.

Owing to a seamless generalization to open-shell systems, ε-SPA^H^M performs well on data sets characterized by a wide variation of charge and spin, for which the traditional structure-based representations commonly fail. However, it suffers from some limitations: (i) its global nature limits transferability,^63^ (ii) it only exploits eigenvalues, despite the availability of additional information (e.g., the eigenvectors and associated electron densities), and (iii) comparing the orbital energies of compounds having different size and composition lacks physical sense.

To address such limitations, in this work, we expand SPA^H^M and build two types of representations exploiting the electron density extracted from the same approximated Hamiltonians. We then bridge the conceptual advantages of both SOAP^16^ and atomic version of SLATM^26^ (aSLATM) to obtain atomic-density overlap fingerprints, SPA^H^M(a), or bond-density based representation, SPA^H^M(b).

The predictive power of SPA^H^M(a,b) is demonstrated on local (atomic) properties such as atomic partial charges, spin densities, and isotropic magnetic shielding on the QM7 data set.^23,64^ We then show the excellent performance of the models on data sets made of a mix of neutral and radical cationic organic molecules and of radical cations of push–pull azoheteroarene-based photoswitches (APSs). These results importantly highlight the possibility of achieving fast and efficient predictions of chemical properties sensitive to the electronic structure (e.g., charge carrier organic materials or transition-metal-catalyzed reaction steps).

## 2 Theory

**Figure 1:**
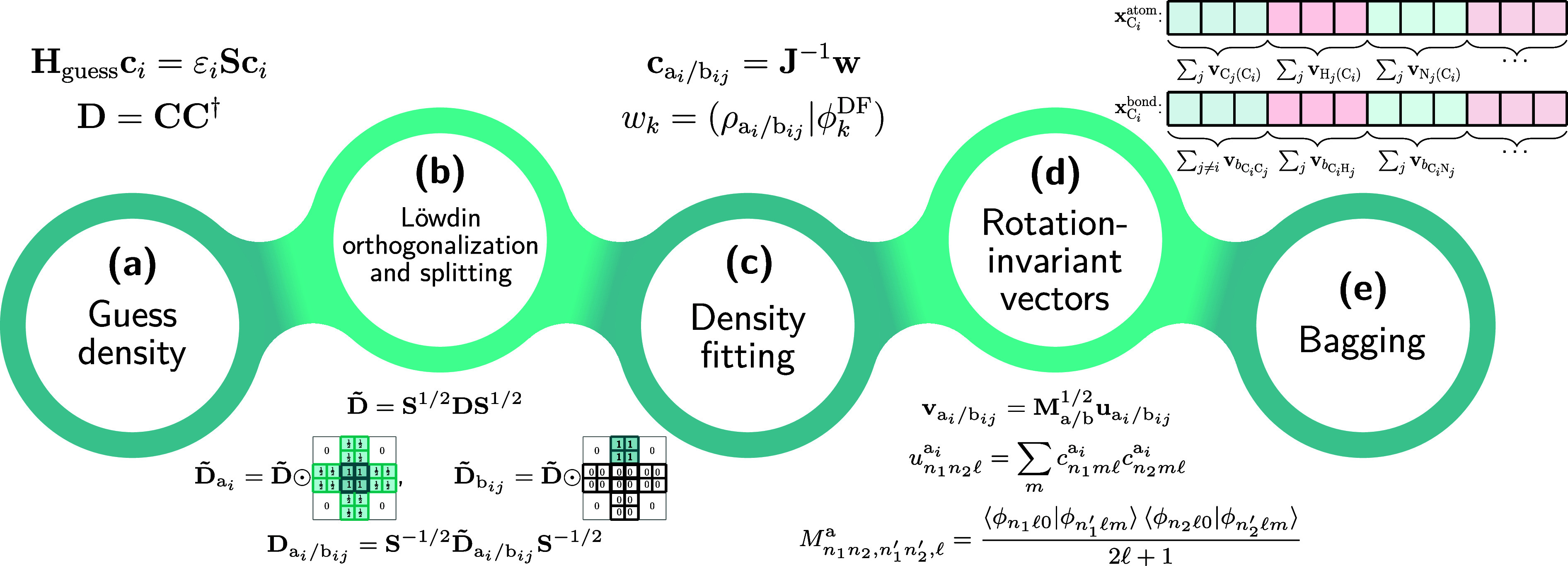
Scheme illustrating the steps required to compute SPA^H^M(a) and SPA^H^M(b) representations.

This section provides a concise description of the proposed atom-based SPA^H^M(a) and bond-based SPA^H^M(b) models introduced in this work. The general workflow used to generate the representation is sketched in Figure 1, and detailed derivations are shown in Section S1 of Supporting Information.

### 2.1 SPA^H^M(a)

This work extends our former representation built from the eigenvalues of lightweight model Hamiltonians. To achieve locality and transferability, the new representations focus on the eigenvectors of those Hamiltonians. However, to avoid dealing with permutational invariance, instead of the eigenvectors our extension is based on the electron density ρ(**r**) or more specifically the preprocessed density matrix **D** (Figure 1a).

Local representations are designed to encode information about each atom within a molecule into a vector. It is thus natural to build our representation from the atomic electron density ρ_*I*_(**r**) of each atom *I*. Yet, there is no unique way to attribute density to an atom.^65−74^ Given the need for analytical solutions, we choose to define ρ_*I*_(**r**) in the form of a decomposition onto an atom-centered basis set in the spirit of the density-fitting approximation.^75−77^

After performing Löwdin orthogonalization^67^ of atomic orbitals, we obtain a separate density matrix attributed to each atom (Figure 1b, left). We then proceed with the density fitting and can take the coefficients **c**_*I*(*I*)_ of the functions centered on the atom of interest (Figure 1c).

The density fitting step allows one to take into account the contribution of atoms *J* ≠ *I* to the ρ_*I*_(**r**), using the coefficients **c**_*J*(*I*)_ of decomposition of ρ_*I*_(**r**) centered on nucleus *J*, thus implicitly including bonding information. The detailed description and comparison with other atomic partitioning schemes is provided in Section S4. Note that in order to include bonding information explicitly, the proposed approach can be generalized to obtain density matrices attributed to each bond (Section 2.2).

The vector of coefficients **c**_*I*_ is not rotationally invariant and hence cannot be directly exploited as a representation. The next step corresponds to the construction of a symmetry-adapted vector **v**_*I*_ (Figure 1d).

Inspired by the SOAP kernel,^16^ we compute the similarity between two atoms *A* and *B* as overlap of ρ_*A*_(**r**) and ρ_*B*_(**r**). To ensure rotational invariance, the overlap is integrated over all possible rotations in 3D space,
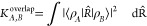
1(To obtain the overlap, the atoms *A* and *B* are virtually placed at the same point of space.) Note that this expression can be generalized to ensure rotational equivariance to learn higher-order tensorial properties in spirit of λ-SOAP.^17^

Each atomic density ρ_*I*_(**r**) is expressed as a sum of terms, centered on the nucleus *I*, hence the overlap kernel can be written as a scalar product of two vectors, *K*_*A*,*B*_^overlap^ = **v**_*A*_^T^**v**_*B*_ (see Section S1A for a detailed derivation of the expression for **v**_*I*_). We disregard the overlap kernel and use **v**_*I*_ as a representation vector of an atom. It provides the following advantages: (i) kernel computation is significantly simplified; (ii) an atomic-density representation can be combined with other vectors; and (iii) the representations can be used with any other kernel function, such as widely used Laplacian and Gaussian kernels.

Another way to obtain a symmetry-adapted vector from **c**_*I*_, reported in the context of ML density functionals,^59^ is to use sum of squares of density-fitting coefficients for each shell. Comparison with our representation is provided in Section S8.

The last step is to construct an atomic representation **x**_*I*_ from the symmetry-adapted vectors **v**_*J*(*I*)_. We regroup all the vectors according to the charge of the nucleus *J* into “bags” of element types, inspired by the construction of aSLATM.^26^ Finally, we sum up the features in each bag to form the final vector. This procedure is illustrated in Figure 1e (top).

### 2.2 SPA^H^M(b)

As discussed in the previous section, the bonding information is included only implicitly into SPA^H^M(a). A complementary approach consists of building an explicit representation for a bond *IJ* by extracting the corresponding density matrix and the density ρ_*IJ*_(**r**) with the Löwdin formalism^67^ (Figure 1b, right).

Using the standard density fitting approach, ρ_*IJ*_(**r**) could be expressed as a sum of terms centered on *I* and *J*, but this would preclude rewriting the kernel as a scalar product and then extracting a representation vector. For this reason, we instead decompose ρ_*IJ*_ onto a basis set centered in the middle of the *IJ* bond (Figure 1c).

Even though most of the information on the bond-density close to nuclei is lost during this procedure, the behavior in the midbond region is well captured. Bond-centered bases are often used to extend atomic-orbital bases for obtaining accurate interatomic potentials^78,79^ but not for density fitting. We thus optimized the basis for each bond present in the data sets studied (involving elements H, C, N, O, F, and S, see Section 5). The basis set construction is described in Section S6A.

Comparison of two bonds *AB* and *CD* involves aligning them along the *z* axis and superimposing their geometrical centers. The similarity is then computed as an overlap of ρ_*AB*_(**r**) and ρ_*CD*_(**r**), integrated over the rotation around the *z* axis (Figure 1d),
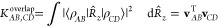
2(See Section S1B for a detailed derivation.)

Simplifications to reduce both the time needed to compute vector **v**_*IJ*_ and its size are possible. For the fitting one can, for instance, use only basis functions with magnetic quantum number *m* = 0 to drop the integration over rotation around the *z* axis or even leave only a single s- or p-orbital. Section S6B illustrates how these simplifications provide a useful compromise for certain data sets.

With the bond-representation vectors {**v**_*IJ*_} at hand, the similarity can be computed between two bonds. While this could be readily used to train bond-property models (e.g., bond dipole moments, dissociation energies), this work focuses on atomic properties requiring one additional step to use the bond vectors and construct an atomic representation.

As for the atom-density representation (Section 2.1), we chose an aSLATM^26^-inspired “bagging” procedure (Figure 1e, bottom). For each atom *A*_*i*_, all the vectors  are grouped according to the element *B* and summed up prior to concatenation. Here, the difference between the bagging of SPA^H^M(a) and SPA^H^M(b) is that the former is sorted according to unique elements (one-body terms in the language of SLATM) and the latter is sorted according to pairs of unique elements (two-body terms). This difference illustrates the complementary focus of the two new variations of SPA^H^M to convert the information from lightweight Hamiltonians into local atomic and bond environments.

## 3 Results and Discussion

### 3.1 Classic Benchmark Data Set: QM7

We assess the learning ability of SPA^H^M(a,b) by predicting two distinct local atomic properties—atomic charges and isotropic magnetic shielding constants—computed for the QM7 database.^23^ For each element (H, C, N, O, and S) and property, a separate kernel ridge regression (KRR) model is trained using its own hyperparameters (see Section 5). Each set was randomly divided into a training and test set (80–20% split).

For each molecule, the LB^80^ guess Hamiltonian paired with a minimal basis set^81^ is diagonalized to obtain the atomic SPA^H^M(a,b) representations following the procedure described in Section 2 and Figure 1. The LB guess was chosen owing to its best performance for the eigenvalue-based SPA^H^M (ε-SPA^H^M).^62^ Comparisons with the Hückel^82,83^ and PBE0^84^ Hamiltonians are provided in Section S7. Briefly, there is a correlation between the quality of the initial guess and the performance of the representation, which opens the way to improving SPA^H^M(a,b) through modifying the underlying guess Hamiltonian.

**Figure 2:**
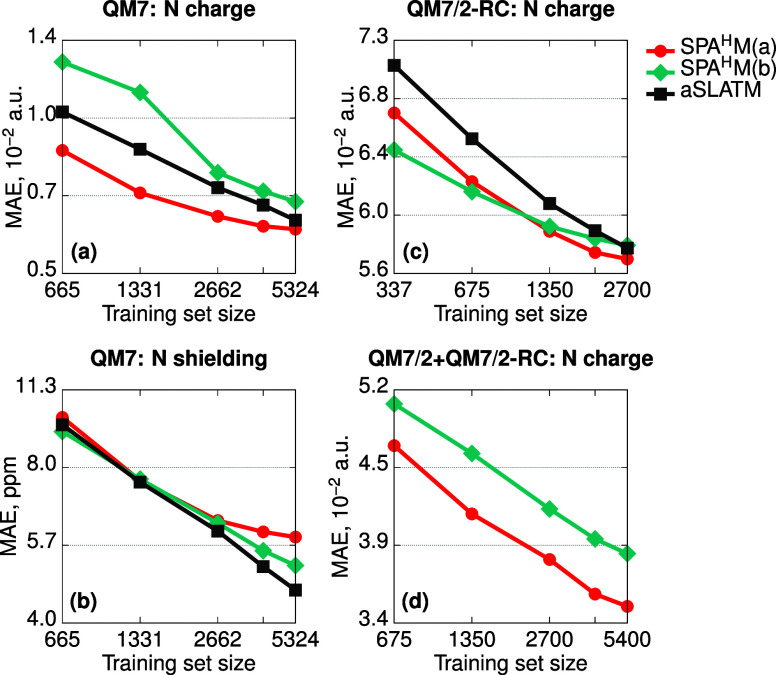
Learning curves for different data sets on the exemplary task of predicting local properties of nitrogen atoms: (a) atomic charges and (b) isotropic magnetic shielding constants for QM7 and atomic charges for (c) radical cations of 3600 QM7 molecules (QM7/2-RC) and (d) mix of 3600 QM7 molecules and 3600 radical cations (QM7/2 + QM7/2-RC). The QM7/2 + QM7/2-RC aSLATM curve is missing since aSLATM is not injective and therefore inappropriate for this data set.

The learning curves of SPA^H^M(a) and SPA^H^M(b) for nitrogen atomic charges are shown in Figure 2a with comparisons with those of aSLATM^26^ (learning curves for other elements and properties are reported in Section S2A). SPA^H^M(a) errors are comparable with those of aSLATM with no clear systematic trend across all the distinct elements (see Section S2A). The generally good performance of SPA^H^M(a) arises from its well-suited atomic-density fingerprints, which encode similar information to atomic charges. Interestingly, the somewhat more sophisticated bond-variant SPA^H^M(b) performs worse than SPA^H^M(a), implying that the bonding information is less relevant for this task. This contrasts with the predictions of isotropic shielding constants (Figure 2b) for which SPA^H^M(b) is systematically superior to SPA^H^M(a) owing to its dependence on the presence of multiple bonds and π-conjugation, which are better captured by the bond density-based model. Yet, for this property neither SPA^H^M(a,b) outperform aSLATM. Specifically, for the hydrogen atom (Section S2A), most frequently analyzed in NMR studies of organic compounds, the SPA^H^M(b) error is ∼1.5 times higher than the aSLATM one.

This result is however not surprising as it was previously demonstrated with ε-SPA^H^M that the strength of the approach lies in capturing the properties of data sets covering a broad range of chemical compositions and electronic structures featuring a variety of charges and spins.^62^ We thus train the model on two databases containing more electronically diverse species. The first one is made of radical cations of 3600 structures randomly selected from QM7 (QM7/2-RC), and the second is a mixture of these 3600 neutral molecules and 3600 radical cations (QM7/2 + QM7/2-RC). The learning curves for nitrogen atomic charge are shown in Figure 2c,d, respectively. For the QM7/2-RC data set, we also predict atomic spin densities (the complete set of learning curves available in Section S2A). The SPA^H^M(a,b) vectors for open-shell molecules are built from concatenation of vectors obtained for α and β densities (see Section S5 for more details).

Figure 2c illustrates the improved performance of SPA^H^M(a,b) with respect to aSLATM, for the difficult task of learning atomic charges of charged species. Thanks to its rooting in the electron density, SPA^H^M is able to capture local changes in the electronic structure. For QM7/2 + QM7/2-RC (Figure 2d), aSLATM cannot be used as it yields the same representation vector for a neutral molecule and its radical cation. On the other hand, SPA^H^M(a,b) seamlessly include the electronic information. The overall prediction errors are approximately averaged errors for the QM7 and QM7/2-RC data sets. However, SPA^H^M(b) is always worse than SPA^H^M(a) for both charges and spins for the same reason as that discussed above.

### 3.2 Tunable Push–Pull Azoheteroarene-Based Dyes

To assess the performance of SPA^H^M(a,b) beyond prototypical molecular examples, we consider a combinatorial database of push–pull APSs^85^, containing 3429 molecules. While this database was originally designed to analyze the tunability of excited states for this class of dyes, we first investigate their hole-carrier properties and train predictive models for the atomic charges and spins of radical cations (APS-RC).

#### 3.2.1 Predicting Hole-Carrier Properties

**Figure 3:**
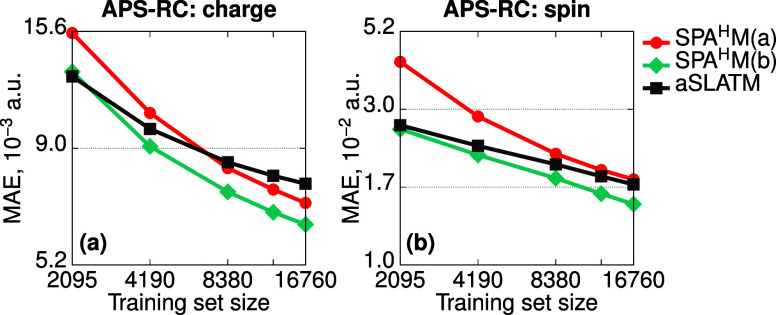
Learning curves of atomic charges and spins of nitrogen for the APS-RC data set. The reference properties are computed at the ωB97X-D/def2-SVP level (see Section 5).

For the APS-RC data set, the learning curves of SPA^H^M(a,b) and aSLATM for nitrogen are shown in Figure 3 (see Figure S2 of Section S2B for other elements). Akin to QM7/2-RC, SPA^H^M(a) performs systematically better than aSLATM but SPA^H^M(b) leads to the lowest errors for this set. The superiority of SPA^H^M(b) can be understood by taking a closer look at the chemical compositions of the two sets. QM7 consists of organic molecules with seven or less heavy atoms. While it contains a large amount of structures with multiple and/or π-conjugated bonds, there are only a few aromatic molecules and thus a restricted number of fragments promoting extensive π-electron delocalization. In contrast, the APS dyes are built from 2 to 4 donor–acceptor aromatic groups interacting through the azo moieties, forming a fairly long π-conjugated scaffold prone to high charge and spin delocalization. The bond-centered representation, which relies upon basis functions with components spatially orthogonal to the bond, is suited to capture these electronic changes. A deeper analysis of an individual molecule is provided in Section S3A.

#### 3.2.2 Predicting Excited-State Properties

Next, we challenged the representations with the APS data set, considering a productive π → π* excited state. In line with the original work,^85^ we focused on learning atomic contributions to the hole and particle densities (computed in the same way as Hirshfeld charges).

**Figure 4:**
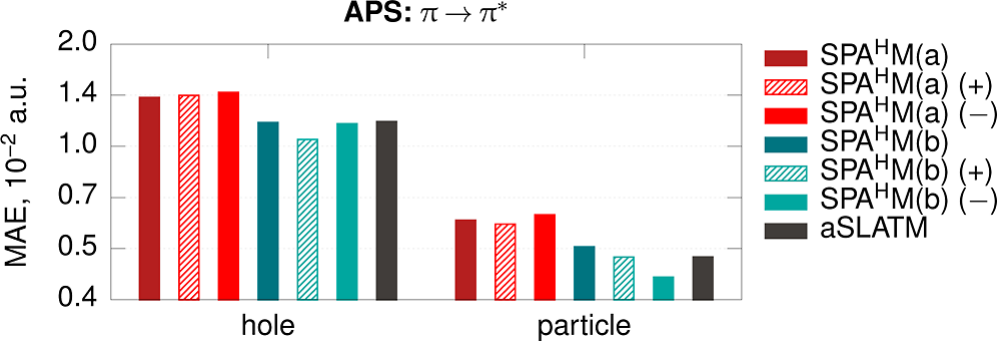
Prediction errors at the full training set for contributions of nitrogen atoms to the hole and particle densities of the productive π → π* state for the APS data set; (+) and (−) indicate SPA^H^M computed for radical cations and anions, respectively. The reference properties are computed with TDDFT at the ωB97X-D/def2-SVP level (see Section 5).

SPA^H^M is computed from a ground-state initial guess; thus, it cannot be expected to predict excited-state properties well. Since the targets are atomic hole and particle contributions, a reasonable approach is to use radical cation and anion densities, respectively, as a starting point. Another choice would be to compute SPAHM from the guess HOMO and LUMO densities, but it is not assessed here. The prediction errors for neutral, cation, and anion SPA^H^M(a,b) and aSLATM for nitrogen are shown in Figure 4 (see Figures S3 and S4 of Section S2B for other elements).

Among the three SPA^H^M density sources, the anion one is systematically the best for the particle contributions and the cation one for the hole contributions, while, as expected, the neutral one is usually the worst.

Only the anion-SPA^H^M contains information on the LUMO, which explains its good performance for the particle contributions (particle density consists of unoccupied orbitals) and low performance for the hole contributions (hole density consists of occupied orbitals; thus, the LUMO information just adds extra noise).

The better performance of cation-SPA^H^M in the case of hole contributions could be explained differently. For open-shell systems, SPA^H^M(a,b) consist of two concatenated vectors constructed from the α- and β-densities. Thus, the full vector implicitly contains the information on the HOMO orbital density, which is removed from the β-vector with respect to α.

In total, for the productive π → π* state of the azo-photoswitches, we found the cation-SPA^H^M(b) and anion-SPA^H^M(b) to be the best for the hole and particle properties, respectively, and expect the same trend for similar excited states. For all the elements in the data set, this approach outperforms aSLATM, which proves that the SPA^H^M family can be useful also for excited-state properties.

#### 3.2.3 Out-of-Sample Prediction

**Figure 5:**
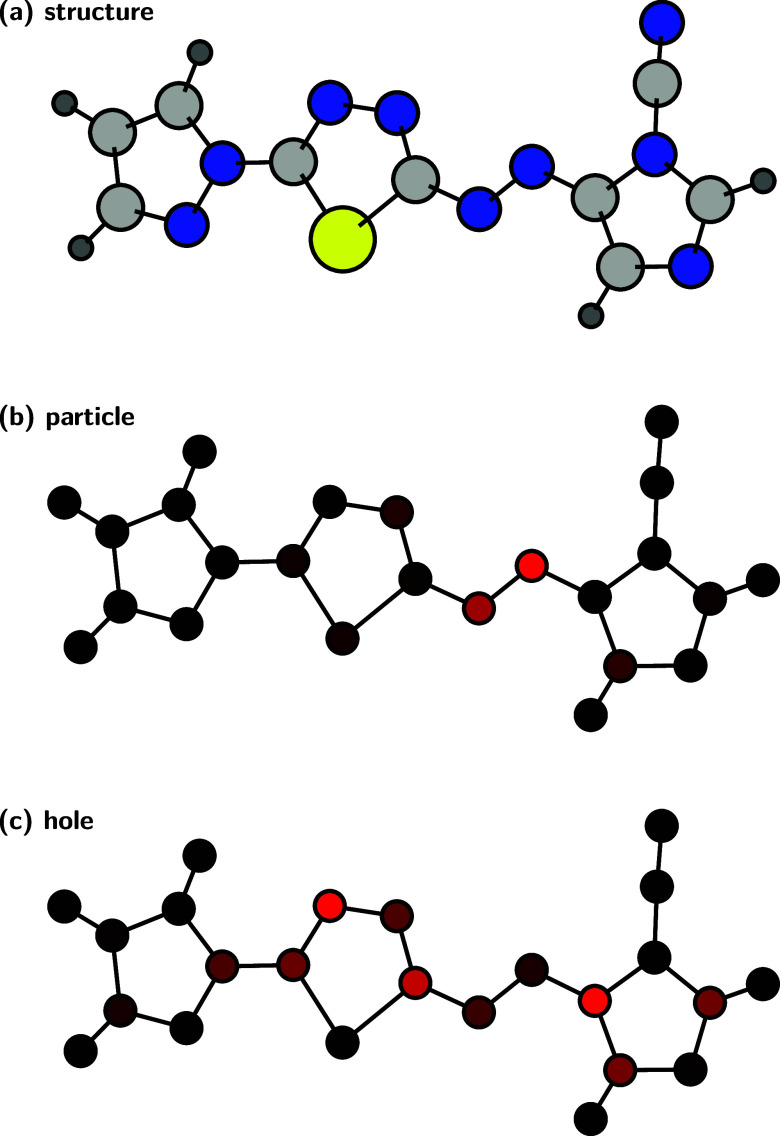
Qualitative picture for (a) an out-of-sample structure: atomic contributions to the (b) particle and (c) hole densities of the productive π → π* state, predicted by SPA^H^M(b). (a) Elements are color-coded by dark gray for H, light gray for C, blue for N, and yellow for S. (b,c) Contribution of each atom is represented by the color intensity from black (0) to red (max. value).

We also predicted hole and particle contributions for an out-of-sample molecule, for which a graphical representation is shown in Figure 5, and the numerical values are provided in Section S3B. This structure is one of several that were excluded from training in the original work^85^ because the computations yielded two almost degenerate π → π* states of mixed character which made it impossible to identify the target state. On the other hand, this makes it a good out-of-sample example because, in contrast to a quantum-chemical computation, the ML model does not know about other excited states and thus can predict the properties of the “correct” state as if it existed.

The predicted values show a picture typical for this excitation:^85,86^ the particle density is mostly localized on the azo group (which makes it much easier to learn than the hole density). Conversely, the hole density is delocalized all over the π-system, its asymmetry showing the push–pull character of the excitation.

### 3.3 Efficiency

To complete this work, we evaluate the efficiency of our models compared to aSLATM, for both the feature vectors generation and kernel computation. Since the training of KRR models consists of kernel matrix inversion, which does not explicitly depend on the representation type, the inversion times are not included.

We randomly selected a subset of 1000 molecules from the QM7 (sub-QM7) and APS (sub-APS, fluorine-containing molecules excluded) databases and generated the SPA^H^M(a), SPA^H^M(b), and aSLATM representations. The user times are reported in Table 1.

**Table 1:** User Times Required to Generate the SPA^H^M(a), SPA^H^M(b), and aSLATM Representations and to Compute the Laplacian Kernel for the Sub-QM7 and Sub-APS Sets (1000 Randomly Selected Molecules)^a^

method	sub-QM7					sub-APS					repr. size, features
	repr., h	kernel, s				repr., h	kernel, s				
		H	C	N	O		H	C	N	S	
SPA^H^M(a)	0.9	**1.37**	**0.75**	**0.07**	**0.06**	4.5	**1.86**	**6.08**	**1.60**	**0.09**	**943**
SPA^H^M(b)	8.2	3.36	1.25	0.14	0.06	42.0	3.54	5.79	1.73	0.05	1328
aSLATM	**0.1**	27.9	10.1	0.34	0.31	**0.2**	53.9	93.3	26.1	0.75	10 808

a The values are averaged over five runs.

SPA^H^M(a,b) computation requires diagonalization (per molecule) and density-fitting (per atom/bond in a molecule) procedures, in the worst case scaling cubically with the number of atoms, resulting in being computationally expensive. Compared to aSLATM, generating SPA^H^M(a) vectors is approximately 9-fold more time-consuming [squared for SPA^H^M(b) relatively] for the sub-QM7 data set. Moving toward more complex systems, i.e. the sub-APS data set, reveals a larger observable time complexity than aSLATM: it took about twice more time to compute the aSLATM representation, compared to QM7, and about five times for either SPA^H^M(a,b). However, the overall speed is implementation-dependent, and efficiency is being addressed in ongoing efforts. Moreover, the simplified bond models discussed in Section S3B open the route for future optimizations of SPA^H^M(b).

We extend the analysis by computing the full Laplacian kernel matrices from these representations (for each element separately); the user times are reported in Table 1. For a fixed set, the theoretical complexity of kernel computation is proportional to the representation vector length (number of features). Since the SPA^H^M(a,b) vectors are ∼10 times more compact than the aSLATM ones, kernel computation for the former is significantly faster, which is especially important for multiple runs needed for a hyperparameter search. While extension of SPA^H^M(a,b) to open-shell systems leads to a two times increase of the vector length, it is still linear with respect to the number of elements in the data set in contrast to the cubic dependency of aSLATM.

Thus, despite the computation of the SPA^H^M(a,b) vectors requiring more time than the aSLATM ones, training the SPA^H^M(a,b) + KRR models is more efficient than aSLATM + KRR. This can be advantageous for molecular dynamics in active learning setups.^87^ We also note that in all cases the prediction time is negligible with respect to TDDFT computations while SPA^H^M(b) shows good results for excited-state properties. Finally, comparison of the original SPA^H^M(b) with simplified versions shows little deterioration of the overall performance and offers promising routes toward more efficient implementations (See Section S6B). Additionally, limiting the extent of the bond-based environments by optimizing the cutoff distance would preclude the computation of distant pairs while maintaining relevant motifs. While being currently under investigation, this effort is expected to significantly reduce run times especially for large systems.

## 4 Conclusions

This work expands our lightweight and efficient eigenvalue SPA^H^M representation into a local electron density-based variant. The adopted strategy extends the class of fingerprints derived from an approximated Hamiltonian with two local density-matrix-based representations: SPA^H^M(a) and SPA^H^M(b), accounting for atom and bond contributions.

Combining strategies inspired from state-of-the-art local representations (i.e., SOAP, aSLATM) while simultaneously encoding electronic information, the SPA^H^M variants show excellent predictive power on local atomic properties (e.g., atomic charges, atomic spin density, and isotropic magnetic shielding) of neutral and charged species for both the prototypical QM7 and more challenging (azoheteroarene-based dyes) sets. SPA^H^M(a,b) were shown to outperform aSLATM for predicting properties of cationic species generated from the QM7 database as well as for those of highly conjugated cationic systems. Validation on the azoheteroarene-based dye database also demonstrated that SPA^H^M(b) is especially adapted to describe changes in electron delocalization typically observed in extended π-conjugated systems.

We note that SPA^H^M(a) and SPA^H^M(b) encode the electronic information while retaining compactness with feature vectors about 4- and 9-fold smaller than aSLATM, respectively. In particular, the size of the representations does not depend on the molecular sizes in the data set (i.e., the system size) but rather on the number of unique elements contained in it. Detailed analysis of the efficiency of the models reveals that this constitutes a significant advantage for kernel construction.

Overall, the proposed representations afford a transferable (local) and efficient alternative in QML for the prediction of various electronic-state properties. We also expect the new SPA^H^M variants to provide a powerful and chemically intuitive framework for the prediction of properties of chemical reactions, which require a bond-focus^88^ as found in SPA^H^M(b), and for the description of molecular properties for which geometrical structures do not inherently coincide with electronic structures (e.g., organic electronic materials).

## 5 Methods

The codes used in this paper are available on a dedicated GitHub repository at https://github.com/lcmd-epfl/SPAHM-RHO and on Q-stack, a broader package for custom quantum-chemical routines to promote QML, at https://github.com/lcmd-epfl/Q-stack.

The initial guess densities were obtained in a minimal basis (MINAO^81^) using the LBm potential.^62,80^ (Comparison with the Hückel^82,83^ and PBE0^84^ potentials is provided in Section S7.) To construct the SPA^H^M(a) representation, the cc-pVDZ/JKFIT^89,90^ atom-centered density fitting basis was used. To construct the SPA^H^M(b) representation, a bond-centered density fitting basis was optimized, the procedure is described in Section S6A. The QML^91^ and TENSOAP (SOAPFAST)^92^ packages were used to construct the aSLATM^26^ and SOAP^16,17^ representations, respectively. The KDFA^59^ representation, also used for comparison, has been reimplemented by us based on the LB guess.

In this work, three molecular data sets were used. They were divided into atomic data sets for each element and randomly split into training and test sets (80–20%): (i) QM7^23^ (7165 neutral organic molecules containing 61 959 H, 35 761 C, 6655 N, 5978 O, and 297 S); (ii) QM7/2-RC (radical cations of 3600 randomly selected structures from QM7, containing 31 195 H, 17 946 C, 3375 N, 3020 O, and 152 S); (iii) APS and (iv) APS-RC (3429 azo-photoswitches,^85^ containing 29 526 H, 39 551 C, 20 951 N, 1053 O, 741 F, and 3337 S, and the corresponding radical cations).

The atomic charges and spins and/or hole and particle contributions were computed using dominant Hirshfeld partitioning^68^ at the PBE0^84^/cc-pVQZ^93,94^ level for the QM7^23^ and QM7/2-RC data sets and at the ωB97X-D^95^/def2-SVP^96^ level for the APS^85^ and APS-RC data sets. The excited-state properties were computed with TDDFT within the Tamm–Dancoff approximation.^97^ The isotropic shielding constants were computed at the PBE^98,99^/cc-pVDZ^93^ level. All quantum-chemical computations were made with PySCF 2.0.^90,100^

For each data set, element, property, and representation, a separate KRR model is trained using its own hyperparameters. The hyperparameters (kernel type, kernel width, regularization) were optimized with a grid search using a 5-fold cross-validation procedure, and the learning curves were computed with random subsampling (5 times per point). The optimization and regression codes use the NumPy^101^ and scikit-learn^102^ Python libraries. The optimal hyperparameters can be found in the GitHub repository (https://github.com/lcmd-epfl/SPAHM-RHO) as well as in Materials Cloud (10.24435/materialscloud:1g-w5) together with the learning curves.

## Data Availability

The data and the model that support the findings of this study are freely available in Materials Cloud (10.24435/materialscloud:1g-w5). The code is available in Q-stack (https://github.com/lcmd-epfl/Q-stack) and as a separate GitHub repository at https://github.com/lcmd-epfl/SPAHM-RHO.

## Supporting Information Available

Detailed derivation of SPA^H^M(a,b) overlap kernels; learning curves for all the elements; predictions for the APS-RC and APS out-of-sample system; comparisons of guess Hamiltonians, different atom-density-based models, and generalizations to open-shell systems; SPA^H^M(b) basis set choice discussion; and comparison with the KDFA^59^ representation (PDF)
