# Supplementary material for: SPAHM(a,b): Encoding the Density Information from Guess Hamiltonian in Quantum Machine Learning Representations
Source: J Chem Theory Comput. 2024 Jan 16;20(3):1108–17. doi: 10.1021/acs.jctc.3c01040 (PMC10867806; doi:10.1021/acs.jctc.3c01040)
Supplement: Supplementary file 1 — ct3c01040_si_001.pdf [file ct3c01040_si_001.pdf]

Supplementary Information

SPA^HM(a,b): Encoding the density information from guess Hamiltonian in quantum machine learning representations

Ksenia R. Briling,[1] Yannick Calvino Alonso,[1] Alberto Fabrizio,[1,2] and Clemence Corminboeuf[1,2,a)]

[1)] *Laboratory for Computational Molecular Design, Institute of Chemical Sciences and Engineering, École Polytechnique Fédérale de Lausanne, 1015 Lausanne, Switzerland*

[2)] *National Centre for Computational Design and Discovery of Novel Materials (MARVEL), École Polytechnique Fédérale de Lausanne, 1015 Lausanne, Switzerland*

(Dated: 5 January 2024)

CONTENTS

S1. Derivation of the SPA^HM(a,b) overlap kernels S2
A. Atom density [SPA^HM(a)] S2
B. Bond density [SPA^HM(b)] S3

S2. Learning curves S4
A. QM7 and its derivatives S4
B. APS-RC and APS S5

S3. Out-of-sample system S8
A. APS-RC S8
B. APS S8

S4. Comparison of different atom-density-based models S9

S5. Generalization to open-shell systems S11

S6. Basis set for the bond-density-based representation S12
A. Optimization S12
B. Simplified models S14

S7. Effect of the Hamiltonian S16

S8. Comparison with the KDFA representation S17

References S17

[a)] Electronic mail: clemence.corminboeuf@epfl.ch

S1. DERIVATION OF THE SPA^{H}M(A,B) OVERLAP KERNELS

A. Atom density [SPA^{H}M(a)]

Let us consider two atoms, A and B. Each atomic density $\rho_I(\mathbf{r})$ is represented as a linear combination of atom-centered spherical Gaussian basis functions $\{\phi_{n\ell m}\}$, labeled with their radial channel number n and angular ℓ and magnetic m quantum numbers,

$$\rho_A(\mathbf{r}) = \sum_{n\ell m} c^A_{n\ell m}\phi_{n\ell m}(\mathbf{r}), \quad \rho_B(\mathbf{r}) = \sum_{n'\ell' m'} c^B_{n'\ell' m'}\phi_{n'\ell' m'}(\mathbf{r}), \tag{S1}$$

and each nucleus is virtually positioned at the origin.

The overlap kernel $K^{\text{overlap}}_{A,B}$ between atoms A and B is the squared overlap of ρ_A and ρ_B averaged over all possible relative orientations $\hat{R}$,

$$K^{\text{overlap}}_{A,B} = \frac{1}{8\pi^2}\int \left|\langle\rho_A|\hat{R}|\rho_B\rangle\right|^2 \mathrm{d}\hat{R} = \frac{1}{8\pi^2}\int \left|k_{AB}(\hat{R})\right|^2 \mathrm{d}\hat{R}. \tag{S2}$$

For a given orientation, the overlap $k_{AB}(\hat{R})$ is

$$\begin{aligned} k_{AB}(\hat{R}) = \langle\rho_A|\hat{R}|\rho_B\rangle &= \int \rho_A(\mathbf{r})\hat{R}\,\rho_B(\mathbf{r})\,\mathrm{d}^3\mathbf{r} \\ &= \sum_{n\ell m} c^A_{n\ell m} \sum_{n'\ell' m'} c^B_{n'\ell' m'}\,\langle\phi_{n\ell m}|\hat{R}\,\phi_{n'\ell' m'}\rangle \\ &= \sum_{n\ell m} c^A_{n\ell m} \sum_{n'\ell' m'} c^B_{n'\ell' m'}\left\langle \phi_{n\ell m}\,\middle|\,\sum_{m''}\phi_{n'\ell' m''}D^{\ell'}_{m''m'}(\hat{R})\right\rangle \\ &= \sum_{\ell}\sum_{nm}\sum_{n'm'} c^A_{n\ell m}c^B_{n'\ell m'}A^{\ell}_{nn'}D^{\ell}_{mm'}(\hat{R}), \end{aligned} \tag{S3}$$

where $\mathbf{D}$ are Wigner D-matrices for *real* spherical harmonics[S1] and $A^{\ell}_{nn'} = \langle\phi_{n\ell m}|\phi_{n'\ell m}\rangle\ \forall m$.

The kernel becomes

$$\begin{aligned} K^{\text{overlap}}_{A,B} &= \frac{1}{8\pi^2}\int \Big|\sum_{\ell}\sum_{nm}\sum_{n'm'} c^A_{n\ell m}c^B_{n'\ell m'}A^{\ell}_{nn'}D^{\ell}_{mm'}(\hat{R})\Big|^2 \mathrm{d}\hat{R} \\ &= \frac{1}{8\pi^2}\sum_{\substack{\ell_1\, n_1m_1\, n'_1m'_1 \\ \ell_2\, n_2m_2\, n'_2m'_2}} c^A_{n_1\ell_1m_1}c^B_{n'_1\ell_1m'_1}c^A_{n_2\ell_2m_2}c^B_{n'_2\ell_2m'_2}A^{\ell_1}_{n_1n'_1}A^{\ell_2}_{n_2n'_2}\cdot\int D^{\ell_1}_{m_1m'_1}(\hat{R})\,D^{\ell_2}_{m_2m'_2}(\hat{R})\,\mathrm{d}\hat{R}. \end{aligned} \tag{S4}$$

Thanks to orthogonality of the real Wigner D-matrices,[S1] *i.e.*,

$$\int D^{\ell_1}_{m_1m'_1}(\hat{R})\,D^{\ell_2}_{m_2m'_2}(\hat{R})\,\mathrm{d}\hat{R} = \frac{8\pi^2}{2\ell_1+1}\,\delta_{\ell_1\ell_2}\delta_{m_1m_2}\delta_{m'_1m'_2}, \tag{S5}$$

the kernel is further simplified to

$$K^{\text{overlap}}_{A,B} = \sum_{\ell}\sum_{\substack{n_1n'_1\\n_2n'_2}} \underbrace{\left(\sum_m c^A_{n_1\ell m}c^A_{n_2\ell m}\right)}_{u^A_p}\underbrace{\left(\frac{A^{\ell}_{n_1n'_1}A^{\ell}_{n_2n'_2}}{2\ell+1}\right)}_{M_{pq}}\underbrace{\left(\sum_m c^B_{n'_1\ell m}c^B_{n'_2\ell m}\right)}_{u^B_q}. \tag{S6}$$

With $p = (n_1, n_2, \ell), q = (n'_1, n'_2, \ell)$ it can be rewritten as a dot product

$$K^{\text{overlap}}_{A,B} = \sum_{pq} u^A_p M_{pq} u^B_q = \mathbf{u}^{\intercal}_A\mathbf{M}\mathbf{u}_B = (\mathbf{M}^{1/2}\mathbf{u}_A)^{\intercal}(\mathbf{M}^{1/2}\mathbf{u}_B) = \mathbf{v}^{\intercal}_A\mathbf{v}_B, \tag{S7}$$

where $\mathbf{v}_I$ is the representation of an atomic electron density $\rho_I(\mathbf{r})$ and is an analog of the power spectrum of atomic neighbor density.[S2]

B. Bond density [SPA^{H}M(b)]

Now let us consider two bonds, AB and XY. The (Löwdin) bond densities $\rho_{AB}(\mathbf{r})$ and $\rho_{XY}(\mathbf{r})$ are decomposed onto basis sets centered in the middle of each bond,

$$\rho_{AB}(\mathbf{r}) = \sum_i c_i \phi_i(\mathbf{r}), \quad \rho_{XY}(\mathbf{r}) = \sum_j c_j \phi_j(\mathbf{r}), \tag{S8}$$

where a function ϕ_i is defined by a radial channel number n_i and angular ℓ_i and magnetic m_i quantum numbers. Both bonds are aligned along the z-axis and their midpoints are put at the origin.

The overlap kernel $K^{\text{overlap}}_{AB,XY} = \mathcal{I}_1$ between the two bonds AB and XY is defined as a overlap integral $\mathcal{I}_2(\varphi)$ squared averaged over rotations $\hat{\varphi}_z$ around the z-axis,

$$\mathcal{I}_1 = \frac{1}{2\pi}\int_0^{2\pi} \mathrm{d}\varphi \left|\langle \rho_{AB}|\hat{\varphi}_z|\rho_{XY}\rangle\right|^2 = \frac{1}{2\pi}\int_0^{2\pi} \mathrm{d}\varphi \left|\mathcal{I}_2(\varphi)\right|^2. \tag{S9}$$

With the decomposition (S8), the overlap integral $\mathcal{I}_2(\varphi)$ is rewritten with overlap of the basis functions,

$$\mathcal{I}_2(\varphi) = \langle \rho_{AB}|\hat{\varphi}_z|\rho_{XY}\rangle = \sum_{ij} c_i c_j \langle \phi_i|\hat{\varphi}_z|\phi_j\rangle = \sum_{ij} c_i c_j \mathcal{I}_3^{ij}(\varphi), \tag{S10}$$

as well as the kernel $\mathcal{I}_1$,

$$\mathcal{I}_1 = \frac{1}{2\pi}\sum_{iji'j'} c_i c_j c_{i'} c_{j'} \int \mathcal{I}_3^{ij}(\varphi)\mathcal{I}_3^{i'j'}(\varphi)\,\mathrm{d}\varphi = \sum_{iji'j'} c_i c_j c_{i'} c_{j'} \mathcal{I}_6^{iji'j'}. \tag{S11}$$

With the rules for rotation of real spherical harmonics around the quantization axis, the overlap $\mathcal{I}_3(\varphi)$ becomes

$$\mathcal{I}_3^{ij}(\varphi) = \langle \phi_i|\hat{\varphi}_z|\phi_j\rangle = \langle \phi_i|\phi_j\rangle \cos m_j\varphi + \langle \phi_i|\phi_{\bar{j}}\rangle \sin m_j\varphi = S_{ij}\cos m_j\varphi + S_{i\bar{j}}\sin m_j\varphi, \tag{S12}$$

where $\phi_{\bar{j}}$ is the same basis function as ϕ_j but with an opposite phase (*i.e.* $m_j = -m_{\bar{j}}$). The integral over rotation $\mathcal{I}_6$ is simplified to

$$\mathcal{I}_6^{iji'j'} = \delta_{|m_j|,|m_{j'}|}(S_{ij}S_{i'j'} + S_{i\bar{j}}S_{i'\bar{j}'}(1-\delta_{m_j0})), \tag{S13}$$

and the overlap kernel $\mathcal{I}_1$ — to

$$\mathcal{I}_1 = \sum_{iji'j'} c_i c_j c_{i'} c_{j'} \delta_{|m_j|,|m_{j'}|}(S_{ij}S_{i'j'} + S_{i\bar{j}}S_{i'\bar{j}'}(1-\delta_{m_j0})). \tag{S14}$$

When p and q are centered at the same point, $S_{pq} = \delta_{\ell_p\ell_q}\delta_{m_pm_q}A^{\ell_p}_{n_pn_q}$. Thus $\mathcal{I}_1$ is further simplified to

$$\mathcal{I}_1 = \sum_{ii'jj'} \underbrace{(c_i c_{i'} \delta_{|m_i|,|m_{i'}|})}_{u^{AB}_{ii'}} \underbrace{\delta_{\ell_i\ell_j} A^{\ell_i}_{n_in_j} \delta_{\ell_{i'}\ell_{j'}} A^{\ell_{i'}}_{n_{i'}n_{j'}} (\delta_{m_im_j}\delta_{m_{i'}m_{j'}} + \delta_{m_i,-m_j}\delta_{m_{i'},-m_{j'}}(1-\delta_{m_j0}))}_{M_{ii',jj'}} \underbrace{(c_j c_{j'} \delta_{|m_j|,|m_{j'}|})}_{u^{XY}_{jj'}}, \tag{S15}$$

which can be rewritten as a dot product in the same spirit as the atom-density kernel,

$$K^{\text{overlap}}_{AB,XY} = \sum_{ii'jj'} u^{AB}_{ii'} M_{ii',jj'} u^{XY}_{jj'} = \mathbf{u}^{\intercal}_{AB}\mathbf{M}\mathbf{u}_{XY} = (\mathbf{M}^{1/2}\mathbf{u}_{AB})^{\intercal}(\mathbf{M}^{1/2}\mathbf{u}_{XY}) = \mathbf{v}^{\intercal}_{AB}\mathbf{v}_{XY}, \tag{S16}$$

where $\mathbf{v}_{IJ}$ is the representation of a bond density $\rho_{IJ}(\mathbf{r})$.

S2. LEARNING CURVES

A. QM7 and its derivatives

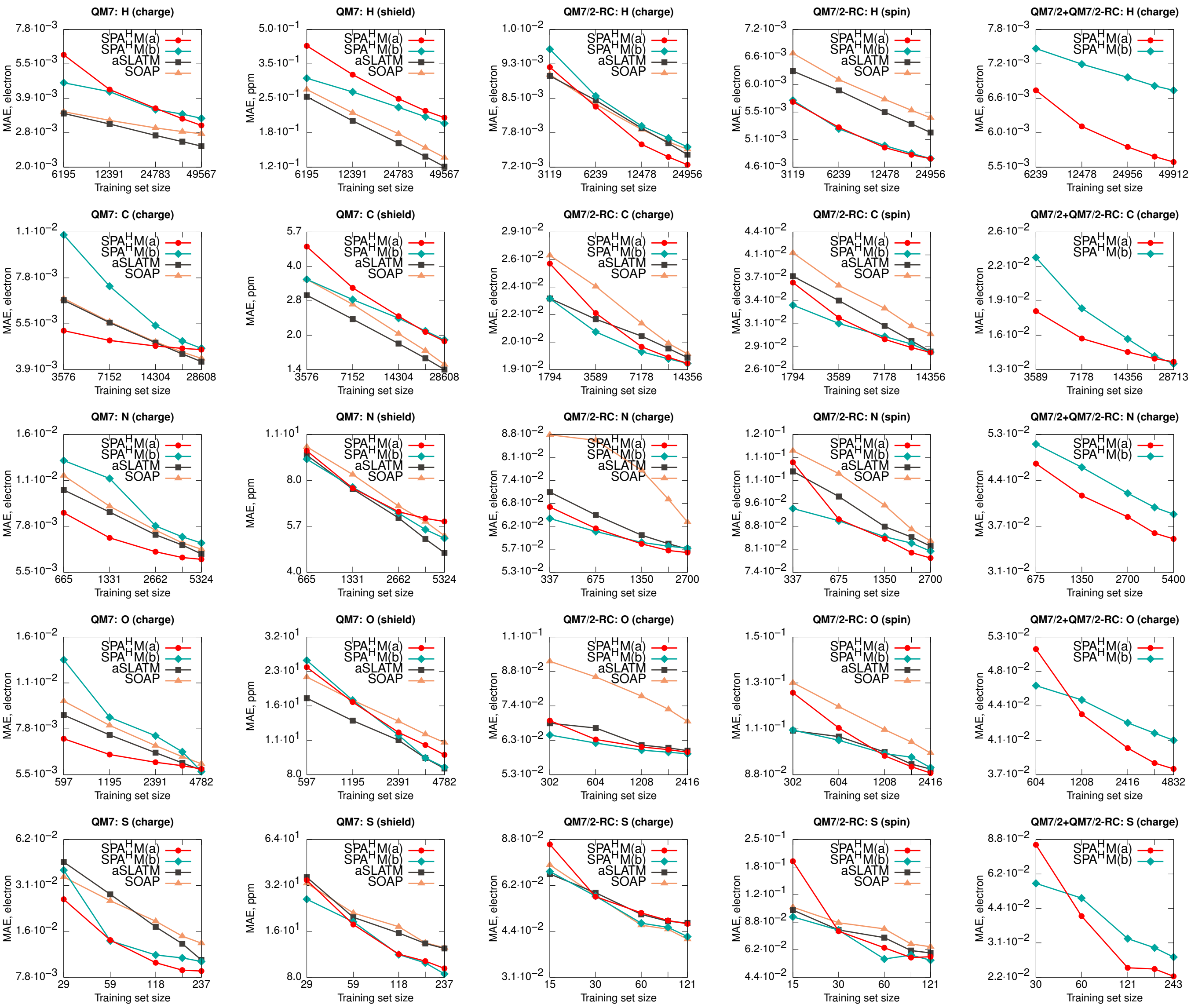

FIG. S1. Learning curves of atomic charges and spins for the QM7, QM7/2-RC, and QM7/2+QM7/2-RC datasets.

B. APS-RC and APS

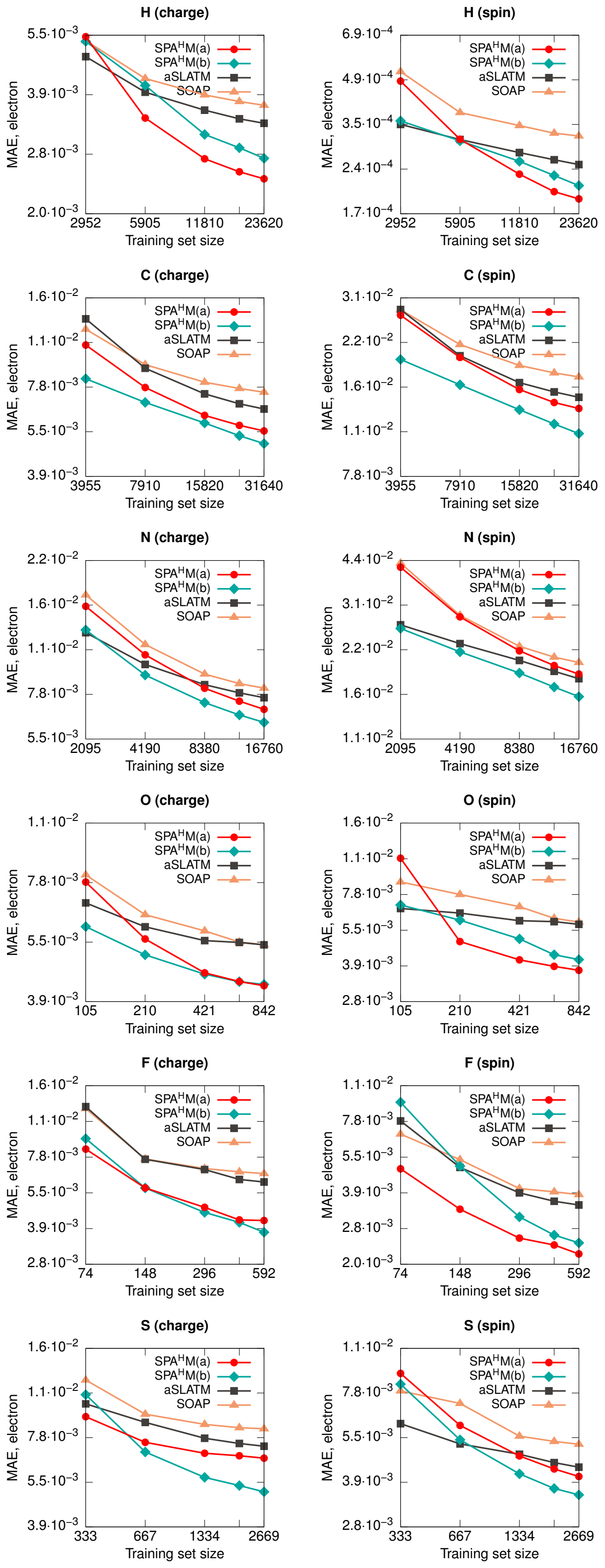

FIG. S2. Learning curves of atomic charges and spins for the APS-RC dataset.

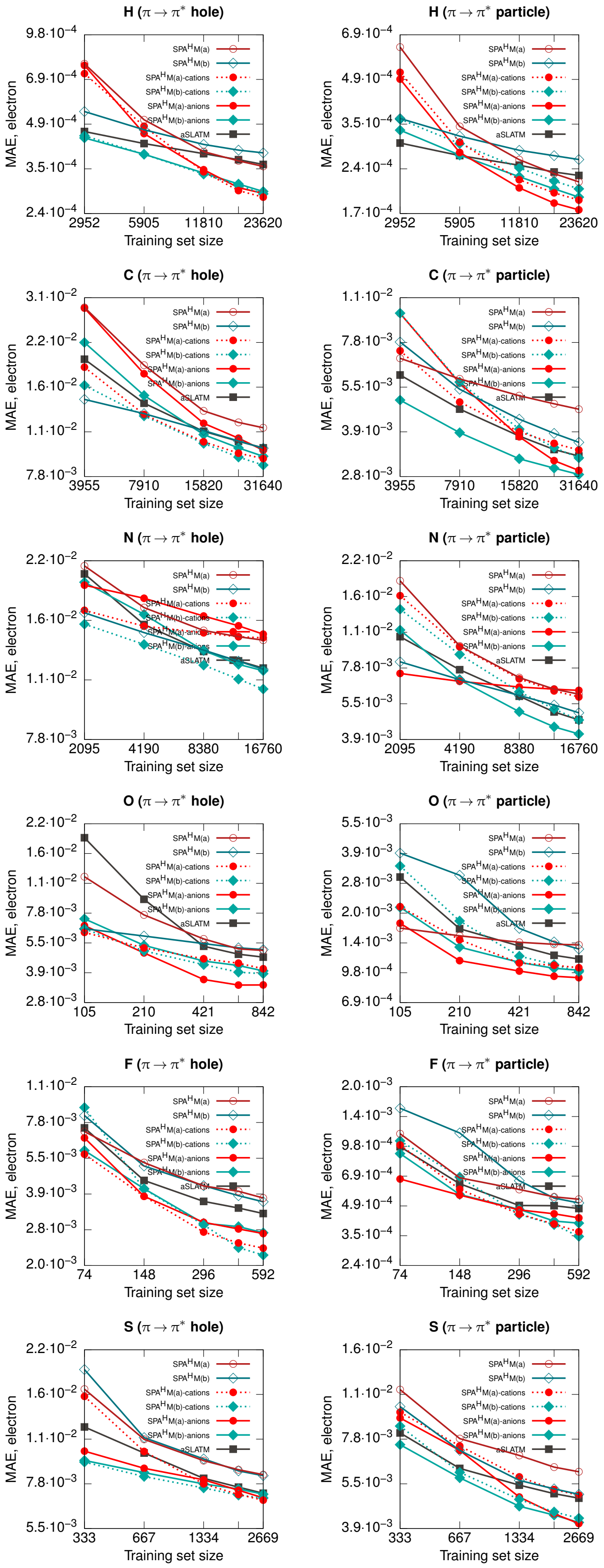

FIG. S3. Learning curves of atomic contributions to the hole and particle densities of the productive $\pi \to \pi^*$ state for the APS dataset; (+) [dashed line] and (−) [solid line] indicate SPA^{H}M computed for radical cations and anions, respectively.

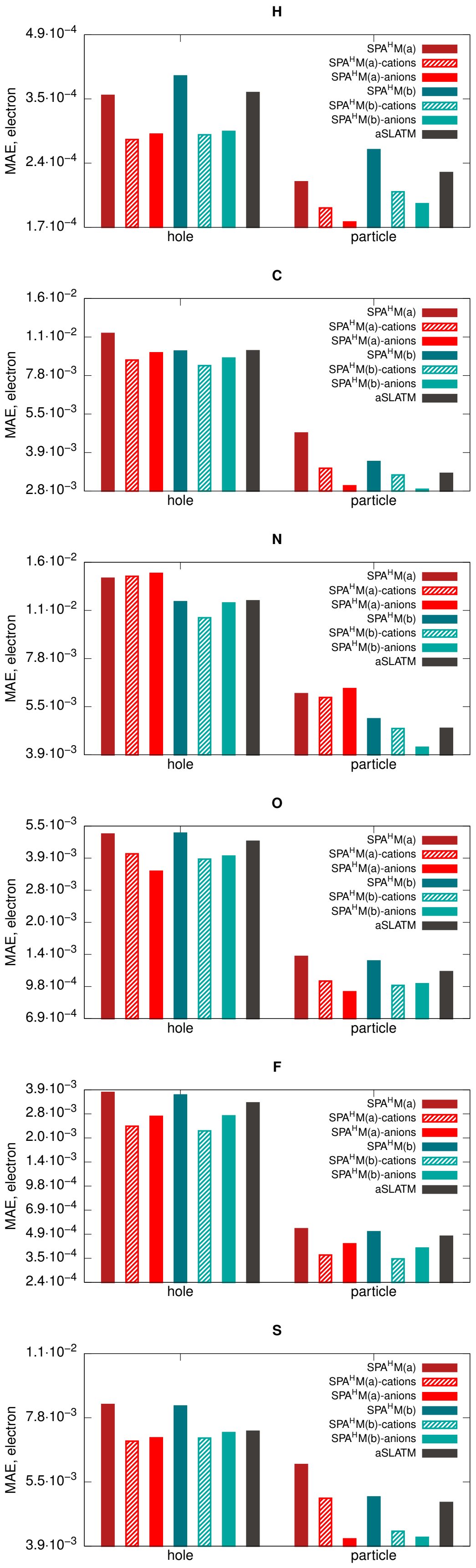

FIG. S4. Histograms of the full training set errors of atomic contributions to the hole and particle densities of the productive $\pi \to \pi^*$ state for the APS dataset; (+) [dashed line] and (−) [solid line] indicate SPA^{H}M computed for radical cations and anions, respectively.

S3. OUT-OF-SAMPLE SYSTEM

A. APS-RC

Analysis of an individual system clearly illustrates the relevance of our models. From the APS-RC dataset we selected an out-of-sample structure and used previously trained SPA^{H}M(a,b) models to predict the atomic charges of its radical cation. Fig. S5 compares the predicted and computed values of atomic charges for a selection of atoms included in the π-conjugated system. For SPA^{H}M(b), the predicted values accurately reproduce the computed ones within 0.01 a.u., thus verifying its performance. However, by taking the changes in atomic charges for all the constituing atoms and summing them up (*i.e.* $\sum_k (q_k^{\mathrm{cation}} - q_k^{\mathrm{neutral}})$) we obtain a total molecular charge ~ 0.9, approximately yielding the removed electron.

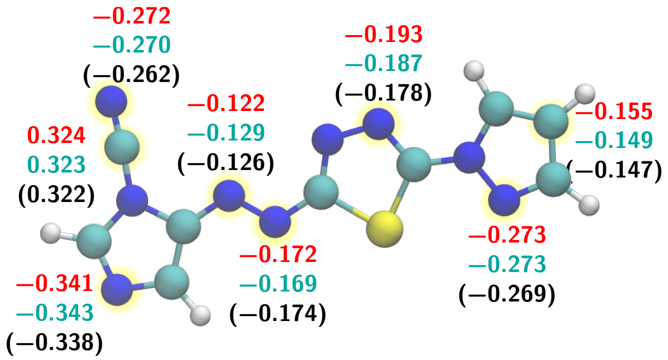

FIG. S5. Predicted by SPA^{H}M(a) (red) and SPA^{H}M(b) (blue) and computed (black) atomic charges for a radical cation of an out-of-sample structure on a selection of atoms (highlighted).

B. APS

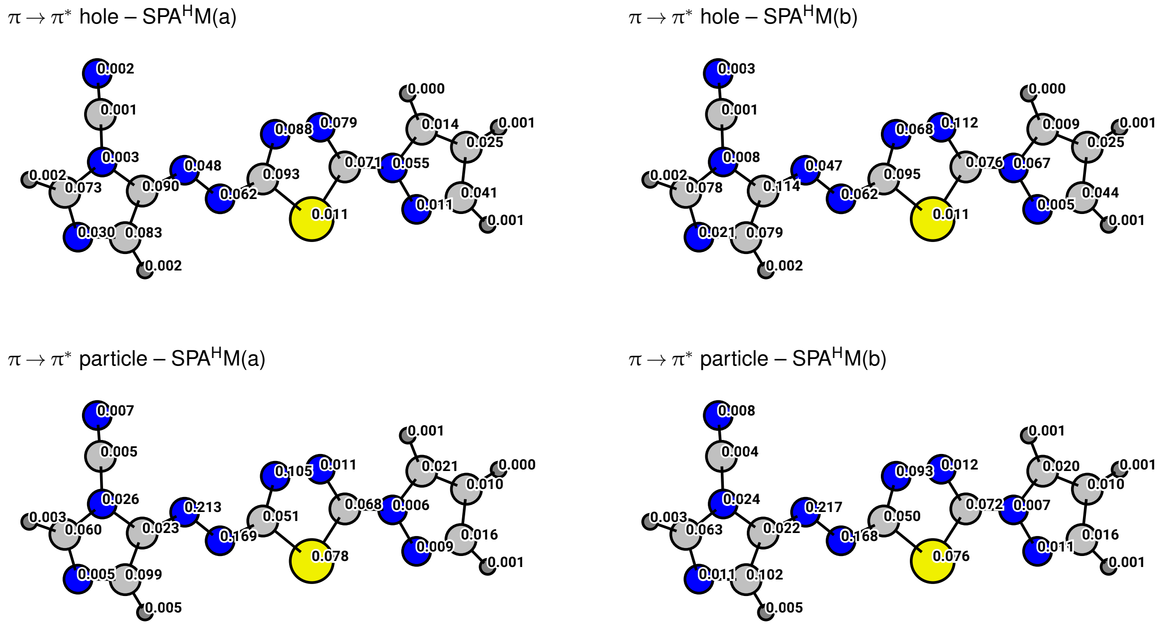

FIG. S6. Predicted by SPA^{H}M(a,b) atomic contributions to the hole and particle densities of the productive $\pi \rightarrow \pi^*$ state for an out-of-sample structure.

S4. COMPARISON OF DIFFERENT ATOM-DENSITY-BASED MODELS

In this section we describe and compare four models used to post-process the guess density matrix. The key elements of all of them are density fitting[S3–S5] (DF), *i.e.* decomposition of the electron density onto an atom-centered basis set,

$$\mathbf{c} = \mathbf{J}^{-1}\mathbf{w}, \quad w_i = \sum_{pq} D_{pq}(\chi_p\chi_q|\phi_i), \tag{S17}$$

where $\mathbf{D}$ is a density matrix, $\{\chi_p\}$ is the atomic orbital basis, $\{\phi_i\}$ is the density-fitting basis, $J_{ij} = (\phi_i|\phi_j)$, and $(\cdots|\cdots)$ is a two-electron integral in chemists' notation, and a subsequent symmetrization described in Sec. S1 A.

- The *pure* model simply consists of fitting the guess density and partitioning of the resulting vector according to the nuclei centers following by symmetrization,

$$\mathbf{D}^{\text{guess}} \xrightarrow{\text{DF}} \mathbf{c} \xrightarrow{\text{part.}} \{\mathbf{c}_I\} \xrightarrow{\text{sym.}} \{\mathbf{v}_I\}, \tag{S18}$$

  (*i.e.* $c_i \in \mathbf{c}_I$ if ϕ_i is centered on the nuclei I).

- The *diff* model consists of the same steps except that the difference between the guess density and the superposition of atomic densities (SAD) is used,

$$\mathbf{D}^{\text{guess}} - \mathbf{D}^{\text{SAD}} \;\Rightarrow\; \mathbf{c} \;\Rightarrow\; \{\mathbf{c}_I\} \;\Rightarrow\; \{\mathbf{v}_I\}. \tag{S19}$$

Both the *short* and *long* models follow the Löwdin population analysis[S6] to partition the molecular density matrix into atomic contributions $\{\mathbf{D}_{(I)}\}$,

$$\mathbf{D}^{\text{guess}} \;\Rightarrow\; \tilde{\mathbf{D}} = \mathbf{S}^{1/2}\mathbf{D}\mathbf{S}^{1/2} \;\Rightarrow\; \{\tilde{\mathbf{D}}_{(I)}\} \;\Rightarrow\; \{\mathbf{D}_{(I)} = \mathbf{S}^{-1/2}\tilde{\mathbf{D}}_{(I)}\mathbf{S}^{-1/2}\}, \tag{S20}$$

where $\mathbf{S}$ is the atomic orbitals overlap matrix. The resulting atomic density matrices $\{\mathbf{D}_{(I)}\}$ are individually subject to density fitting and symmetrization.

- The *long* version includes the coefficients related to other atom centers as a long-range contribution to the atomic density:

$$\mathbf{D}^{\text{guess}} \xrightarrow{\text{Löwdin}} \mathbf{D}^{\text{guess}}_{(I)} \xrightarrow{\text{DF}} \mathbf{c}_{(I)} \xrightarrow{\text{part.}} \{\mathbf{c}_{J(I)}\} \xrightarrow{\text{sym.}} \{\mathbf{v}_{J(I)}\} \quad \forall I. \tag{S21}$$

  To construct the final representation for atom I, the vectors $\{\mathbf{v}_{J(I)}\}$ are grouped according to the nuclear charge of J, summed up, and concatenated, but it is not the only possible way to proceed.

- The *short* version only retains the coefficients directly related to the basis functions centered on the atom of interest,

$$\mathbf{D}^{\text{guess}} \;\Rightarrow\; \mathbf{D}^{\text{guess}}_{I} \;\Rightarrow\; \mathbf{c}_{(I)} \xrightarrow{\text{part.}} \mathbf{c}_{I(I)} \xrightarrow{\text{sym.}} \mathbf{v}_{I(I)} \quad \forall I. \tag{S22}$$

The learning curves for the models are shown on Fig. S7. Overall, the *long* model shows the best overall performance and was selected as default to be used hereinafter.

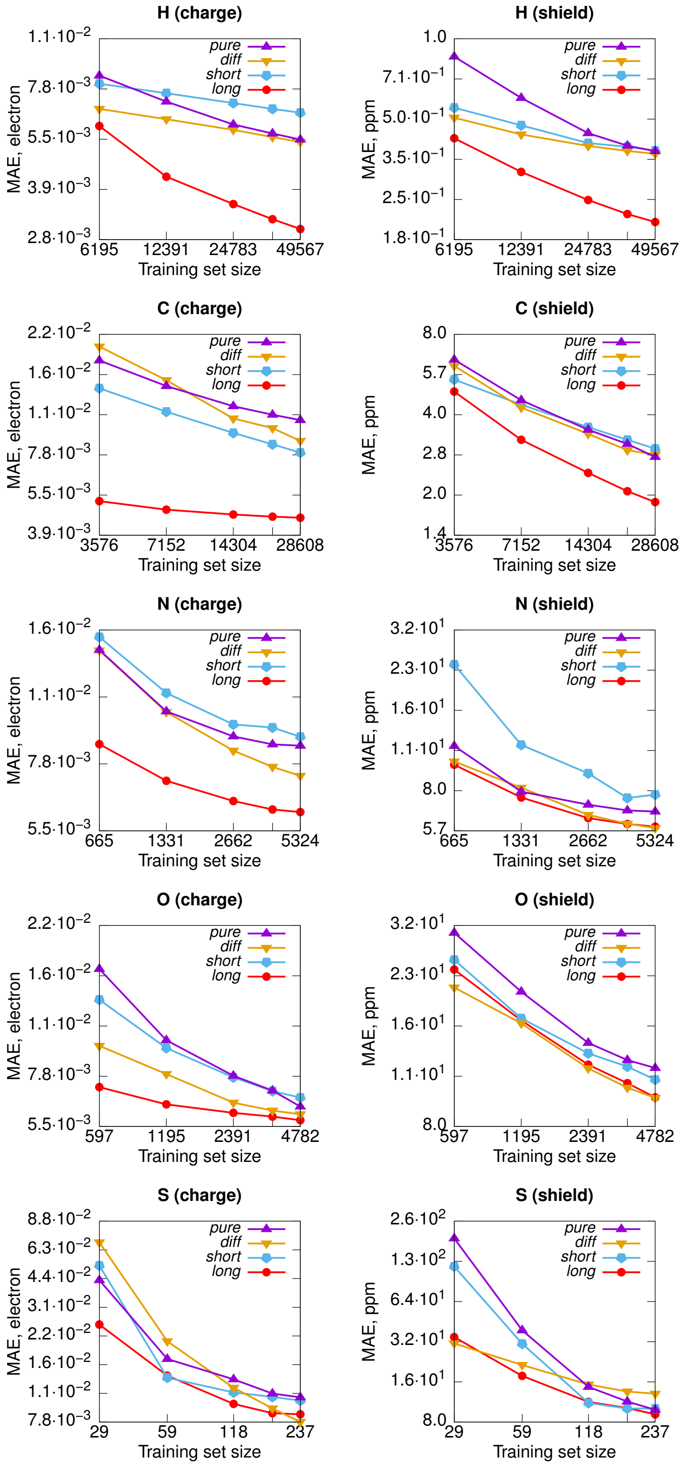

FIG. S7. Learning curves of atomic charges and shielding constants for the QM7 dataset. The color code reflects the different models used to construct the SPA^{H}M(a) representation from the rotationally-invariant vectors.

S5. GENERALIZATION TO OPEN-SHELL SYSTEMS

We considered three ways to generalize the model to open-shell systems:

1) concatenation of representation vectors $\mathbf{x}$ obtained from ρ_α and ρ_β separately ("$\alpha\beta$");

2) representation vector obtained from $\rho = \rho_\alpha + \rho_\beta$, the total electron density as in case of closed-shell systems ("+");

3) concatenation of representation vectors obtained from $\rho = \rho_\alpha + \rho_\beta$ and $\rho_m = \rho_\alpha - \rho_\beta$ separately ("+−").

They were tested on the QM7/2-RC dataset with the SPA^{H}M(b) representation. The results are shown on Fig. S8. As expected, in most cases the "+" model, having no information on the spin density, performed the worst, whereas the "$\alpha\beta$" model showed the best results and was chosen as the default option.

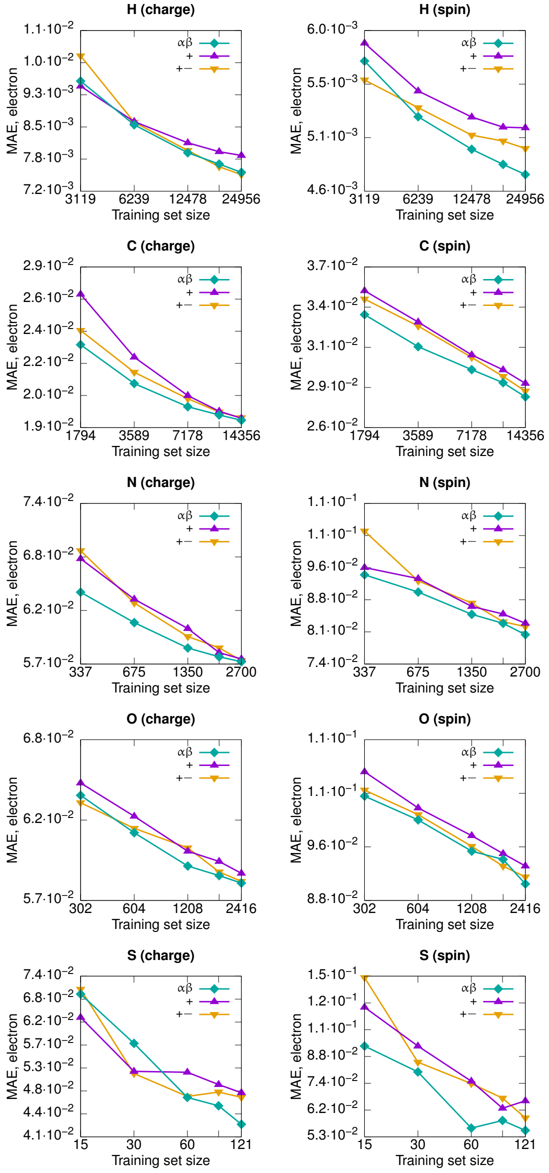

FIG. S8. Learning curves of atomic charges and spins for the QM7/2-RC dataset and the SPA^{H}M(b) representation. The color code reflects the different models used to generalize the representation to open-shell systems.

S6. BASIS SET FOR THE BOND-DENSITY-BASED REPRESENTATION

A. Optimization

The decomposition of the bond density onto a midbond-centered basis set required optimization of a suitable basis. First, we followed the procedure described in Ref. S7 used to optimize a basis to fit the on-top pair density.

For each bond of interest in a molecule, we search for the set of coefficients $\{c_i\}$ that approximates the bond density in the least-squares sense,

$$\rho_{AB}(\mathbf{r}) \approx \sum_i c_i \phi_i(\mathbf{r}), \qquad \mathbf{c} = \mathbf{S}^{-1}\mathbf{b}, \tag{S23}$$

where $\mathbf{S}$ is the overlap matrix, $b_i = \langle \rho_{AB} | \phi_i \rangle$, and the decomposition error is

$$\mathcal{E} = \int \Big(\rho_{AB}(\mathbf{r}) - \sum_i c_i \phi_i(\mathbf{r}) \Big)^2 \, \mathrm{d}^3\mathbf{r} = \langle \rho_{AB} | \rho_{AB} \rangle - \mathbf{b}^\mathsf{T} \mathbf{S}^{-1} \mathbf{b}. \tag{S24}$$

Thus, to optimize the exponents, we minimize the sum of decomposition errors $\mathcal{E}$ for the molecules chosen for the bond of interest. The exponents $\{\alpha_\mu\}$ for all the angular momenta are optimized simultaneously. The exponents are parameterized as $\alpha_\mu = \exp(p_\mu)$, and the first derivatives of the loss functions $\mathcal{E}$ with respect to the exponents are computed as follows,

$$\frac{\partial \mathcal{E}}{\partial \alpha_\mu} = \mathbf{c}^\mathsf{T} \left(\frac{\partial \mathbf{S}}{\partial \alpha_\mu} \mathbf{c} - 2 \, \frac{\partial \mathbf{b}}{\partial \alpha_\mu} \right), \tag{S25}$$

with the overlap integrals and their derivatives taken numerically.

All the bonds were treated separately. For each bond (or atom pair) presented in the QM7 and APS datasets we chose representative molecules containing it (*e.g.*, H_2 for H–H; C_2H_2, C_2H_4, and C_2H_6 for C–C; H_2O and H_2O_2 for H–O), and the sum of the molecular decomposition errors was minimized. The maximum angular momentum ℓ_max and the number of functions n_ℓ for each ℓ were gradually increased and optimized on each step, until addition of further radial functions or angular momenta did not provide any significant decrease of error. The optimized exponents are available separately in `Q-stack` (`https://github.com/lcmd-epfl/Q-stack`).

However, for some of the bonds the fitting errors were huge (up to 20%) due to the fact that largest fraction of the bond density is still localized on participating nuclei, thus the fine-tuning of the fitting basis could not improve much. This could be solved with adding a single Gaussian centered in the midbond as a weight function. Our tests showed that, however the fitting error significantly decreased, the quality of learning was almost the same.

On Fig. S9 we compare the performance of SPA$^\mathrm{H}$M(b) computed using the fully-optimized basis for each bond ("normal") and using the same (C–C) basis for every bond ("same basis"). It is clear that the representation quality does not depend on the exponents of the basis thus their optimization can be omitted. (the role of angular momenta is discussed in Sec. S6 B).

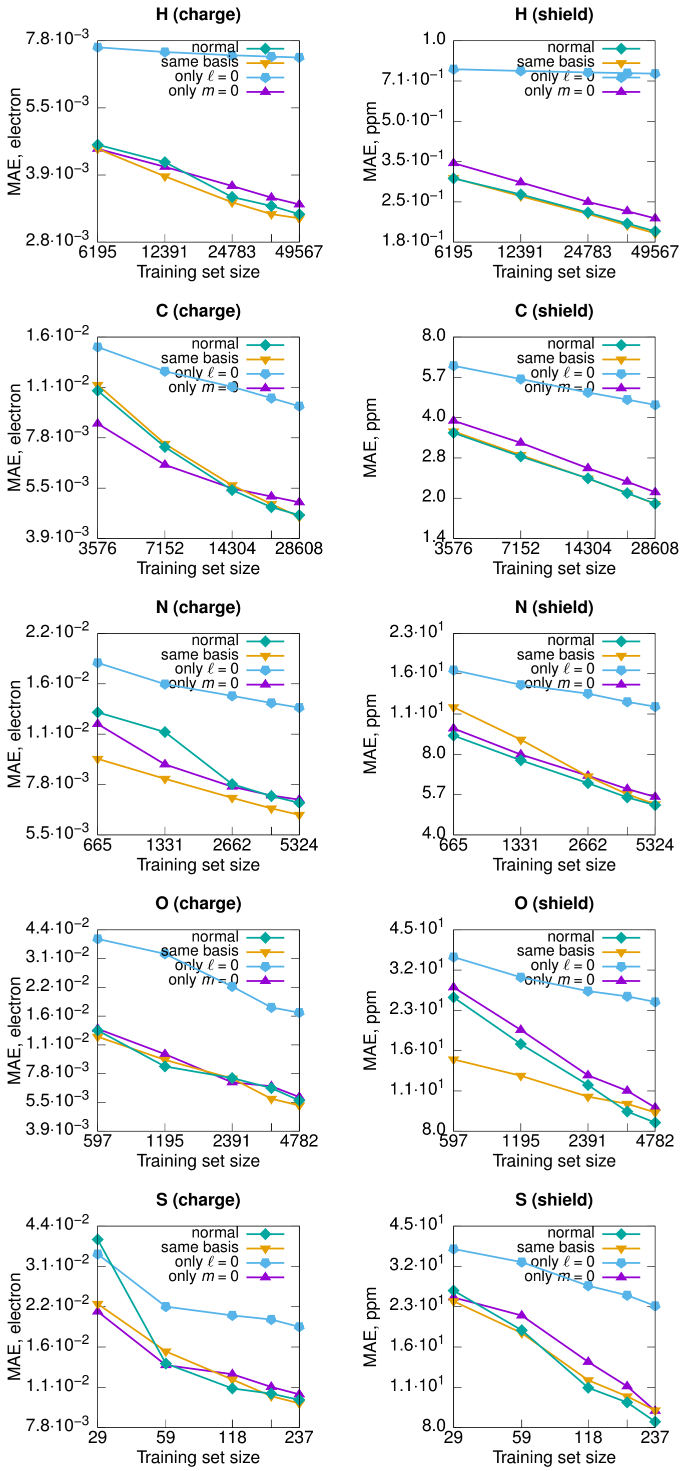

FIG. S9. Learning curves of atomic charges and shielding constants for the QM7 dataset. The color code reflects the different basis sets used to generate the SPA^{H}M(b) representations: "normal": fully-optimized basis for each bond; "same basis": the same (C–C) basis for every bond; "only $\ell = 0$": optimized basis with s-orbitals only; "only $m = 0$": optimized basis with $m \neq 0$ orbitals excluded.

B. Simplified models

We also tested two approaches to simplify the bond-based representation, which reduce the effort for both the two-electron integral evaluation and vector symmetrization.

The first one is to use only the s-orbitals. The learning curves for the QM7 dataset for the representation based on the fully-optimized basis truncated to the functions with $\ell = 0$ are shown on Fig. S9. Its peformance is significantly deteriorated and it is clear that higher angular momenta are necessary.

Another option is to use the orbitals with $m = 0$, *i.e.*, symmetric with respect to rotation around the bond. Then Eq. S15 is simplified to

$$K^{\text{overlap}}_{AB,XY} = \sum_{\ell\ell'} \sum_{\substack{n_1 n_1' \\ n_2 n_2'}} \underbrace{c_{n_1\ell 0} c_{n_1'\ell' 0}}_{u_p^{AB}} \underbrace{A^{\ell}_{n_1 n_2} A^{\ell'}_{n_1' n_2'}}_{M_{pq}} \underbrace{c_{n_2\ell 0} c_{n_2'\ell' 0}}_{u_q^{XY}} . \tag{S26}$$

In the current implementation, the bond density is first projected onto the DF basis set and then rotated so the bond is aligned with the z-axis and the DF coefficients are transformed accordingly. This is why in our tests the density is fitted with the "full" basis set and only the final representation is truncated to have only products of cylindrically-symmetric orbitals.

The learning curves for QM7 and for APS-RC comparing the truncated representation with the full one are shown on Fig. S9 and Fig. S10, respectively. For QM7, the truncated representation yields the same or slightly worse performance, whereas for a more challenging APS-RC it even improves the learning in some cases.

While functions with $\ell = 0$ are not sufficient to construct a good representation, the representation built from $m = 0$ only performs very well on simple organic molecules and at least in the case of the APS-RC dataset the part of the density that seems to be orthogonal to the aromatic ring is well enough captured by *e.g.* d_{z^2}-orbital. This simplification of SPA^{H}M(b) is promising in terms of both performance and potential optimizations and should be studied further.

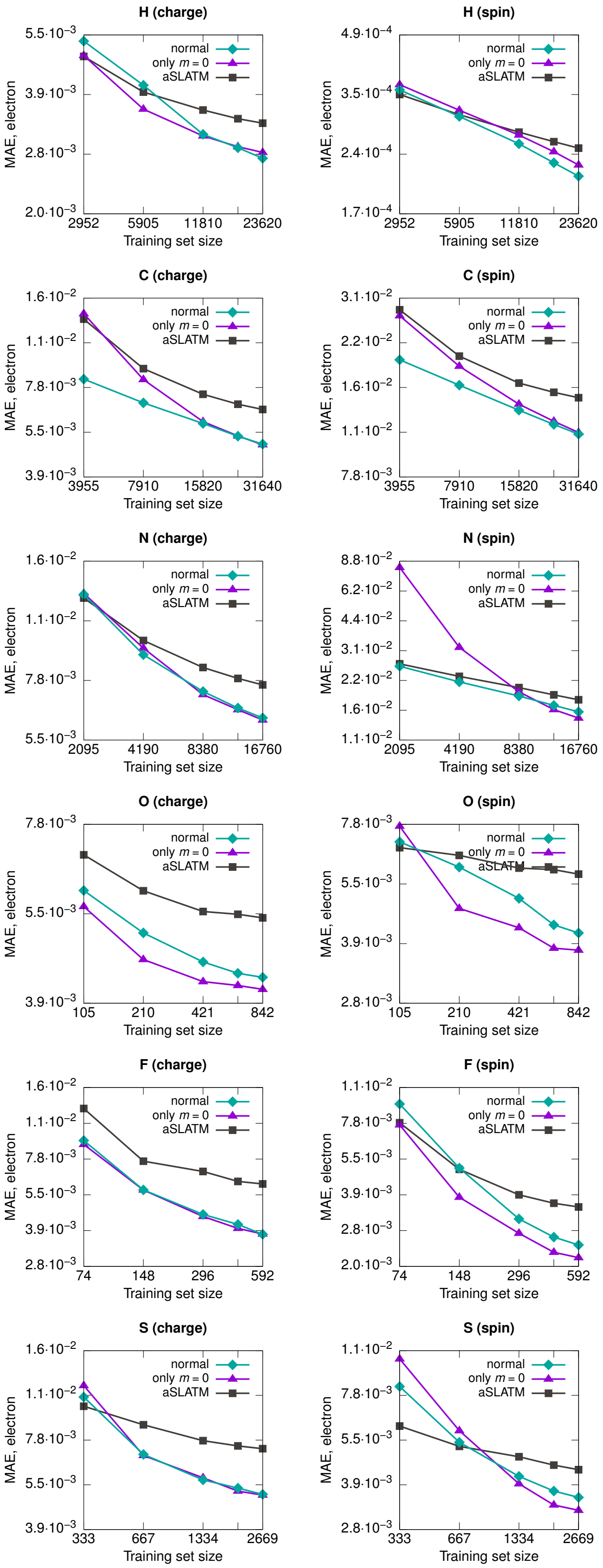

FIG. S10. Learning curves of atomic charges and spins for the APS-RC dataset. The color code reflects the different basis sets used to generate the SPA^{H}M(b) representations: "normal": fully-optimized basis for each bond; "only $m = 0$": optimized basis with $m \neq 0$ orbitals excluded. Learning curves for SLATM are given for comparison.

S7. EFFECT OF THE HAMILTONIAN

We compared the SPA^{H}M(a,b) representations built upon the density matrices obtained from the Hückel guess[S8,S9], the LB[S10] guess (default), and a converged PBE0[S11] computation. The learning curves are shown on Fig. S11. As expected, the worst approximation, the Hückel guess, gives the worst regression results. In contrast to the eigenvalue SPA^{H}M,[S12], the converged density makes the best representation, sometimes overperforming SLATM, which opens the way to improvement of SPA^{H}M(a,b) through improvement of the underlying guess Hamiltonian.

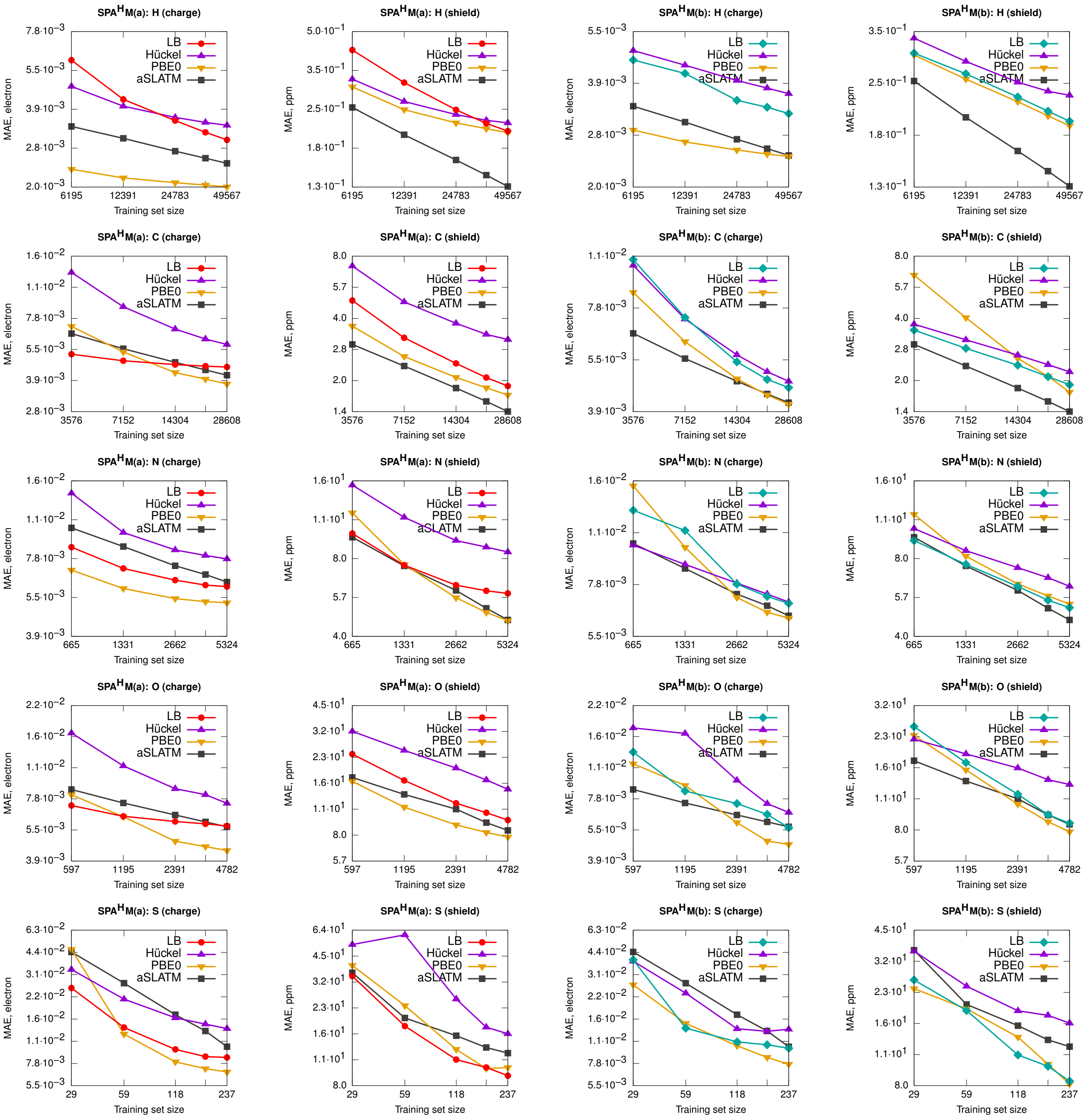

FIG. S11. Learning curves of atomic charges and shielding constants for the QM7 dataset. The color code reflects the different Hamiltonians used to generate the SPA^{H}M(a,b) representations.

S8. COMPARISON WITH THE KDFA REPRESENTATION

Recently the kernel density functional approximation[S13] (KDFA) was proposed, similar in construction to our SPA^{H}M(a) model.

In KDFA, the representation vector for an atom is also built from the density-fitting coefficients of the functions centered on its nucleus. Instead of the coefficients themselves, rotationally-invariant sums $\sum_m |c_{nlm}|^2$ are used. This could be seen as a simplification of Eq. S6 with a combination of Kronecker deltas instead of M_{pq}, omitting the cross-products of different radial basis functions,

$$K_{A,B}^{\mathrm{KDFA}} = \sum_{\substack{\ell \\ n_1 n_1' \\ n_2 n_2'}} \underbrace{\left(\sum_m c^A_{n_1\ell m} c^A_{n_2\ell m}\right)}_{u^A_p} \underbrace{\delta_{n_1 n_2}\delta_{n_1' n_2'}\delta_{n_1 n_1'}}_{M_{pq}} \underbrace{\left(\sum_m c^B_{n_1'\ell m} c^B_{n_2'\ell m}\right)}_{u^B_q} = \sum_{n\ell} \underbrace{\left(\sum_m |c^A_{n\ell m}|^2\right)}_{v^A_p} \underbrace{\left(\sum_m |c^B_{n\ell m}|^2\right)}_{v^B_q}. \tag{S27}$$

The learning curves comparing the performance of the KDFA representation with our *pure* and *long* models (see Sec. S4) are shown of Fig. S12. Overall, the performance of the KDFA representation is close to the *pure* model. However, the *long* model is consistently better, presumably due to inclusion of "long-range" contributions to the atomic density.

REFERENCES

[S1] D. A. Varshalovich, A. N. Moskalev, and V. K. Khersonskii, *Quantum Theory of Angular Momentum* (World Scientific, 1988).
[S2] A. P. Bartók, R. Kondor, and G. Csányi, Phys. Rev. B **87**, 184115 (2013).
[S3] E. J. Baerends, D. E. Ellis, and P. Ros, Chem. Phys. **2**, 41 (1973).
[S4] J. L. Whitten, J. Chem. Phys. **58**, 4496 (1973).
[S5] K. Eichkorn, O. Treutler, H. Öhm, M. Häser, and R. Ahlrichs, Chem. Phys. Lett. **240**, 283 (1995).
[S6] P.-O. Löwdin, J. Chem. Phys. **18**, 365 (1950).
[S7] A. Fabrizio, K. R. Briling, D. D. Girardier, and C. Corminboeuf, J. Chem. Phys. **153**, 204111 (2020).
[S8] R. Hoffmann, J. Chem. Phys. **39**, 1397 (1963).
[S9] S. Lehtola, J. Chem. Theory Comput. **15**, 1593 (2019).
[S10] D. N. Laikov and K. R. Briling, Theor. Chem. Acc. **139**, 17 (2020).
[S11] C. Adamo and V. Barone, J. Chem. Phys. **110**, 6158 (1999).
[S12] A. Fabrizio, K. R. Briling, and C. Corminboeuf, Digital Discovery **1**, 286 (2022).
[S13] J. T. Margraf and K. Reuter, Nat. Commun. **12**, 344 (2021).

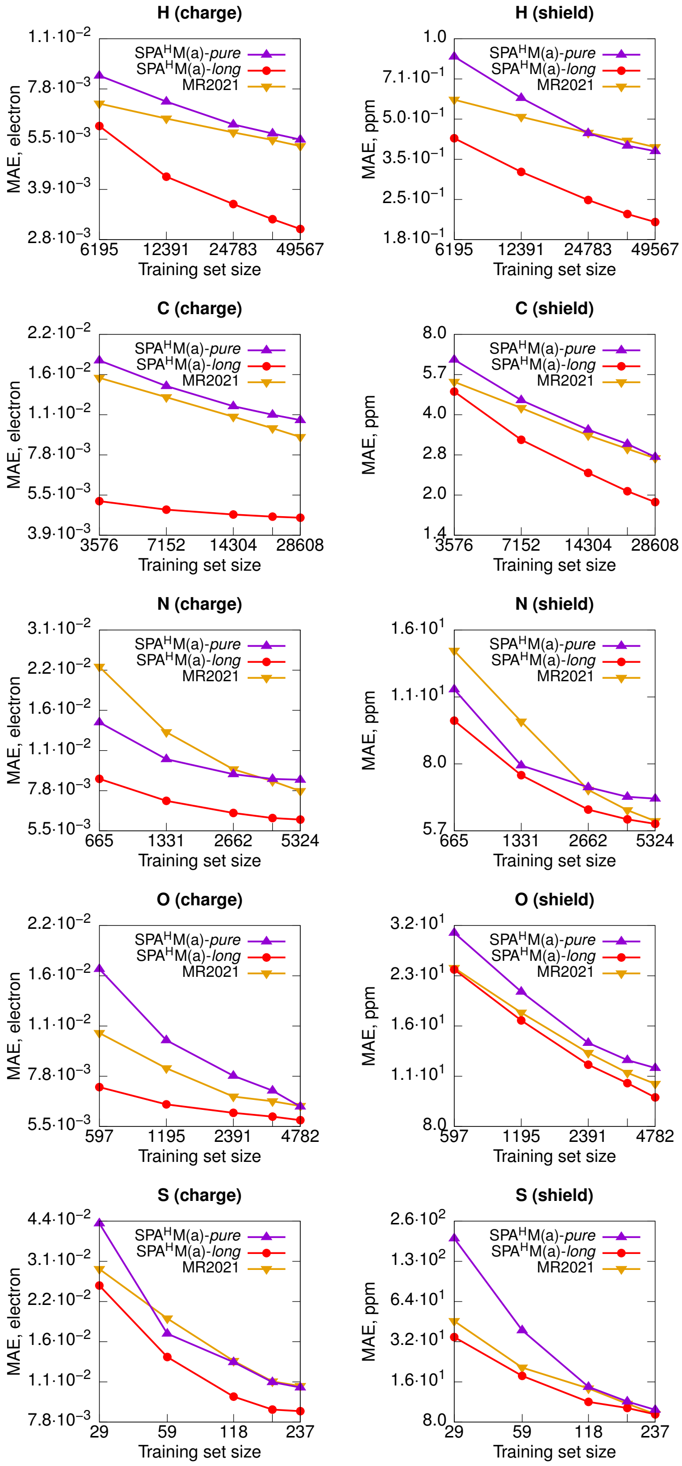

FIG. S12. Learning curves of atomic charges and shielding constants for the QM7 dataset. The color code reflects the different representations. "MR2021" stands for the KDFA[S13] representation.
